# Supplementary material for: Nuclear magnetic resonance for wireless magnetic tracking
Source: Nat Commun. 2025 Dec 2;16:10840. doi: 10.1038/s41467-025-66468-3 (PMC12673108; doi:10.1038/s41467-025-66468-3)
Supplement: Supplementary file 1 — Supplementary_Information [file 41467_2025_66468_MOESM1_ESM.pdf]

Supplementary Materials for

Nuclear Magnetic Resonance For Wireless Magnetic Tracking

M. Efe Tiryaki[1,2,†], Pouria Esmaeili-dokht[1,3,†], Jelena Lazovic[1], Klaas P. Pruessmann[1,4], Metin Sitti[1,5,*]

[1] Physical Intelligence Department, Max Planck Institute for Intelligent Systems, 70569 Stuttgart, Germany
[2] Mechanical Engineering Department, Middle East Technical University, 06800 Ankara, Turkey
[3] Stuttgart Center for Simulation Science, University of Stuttgart, 70569 Stuttgart, Germany
[4] Institute for Biomedical Engineering, ETH Zurich and University of Zurich, 8092 Zurich, Switzerland
[5] School of Medicine and College of Engineering, Koç University, 34450 Istanbul, Turkey
† Equal contributing authors
* Corresponding author: msitti@ku.edu.tr

The PDF file includes:

Supplementary Notes 1 to 15

Supplementary Table 1

Supplementary Figures 1 to 29

Supplementary Notes

1. NMR magnetic sensing: The basis of NMR magnetic sensing resides in the precession of nuclear spins in the presence of a background magnetic field (B0) after excitation by an oscillating external magnetic field. In the presence of B0, the net magnetisation of all the spins will redirect in parallel with the field. By superimposing an oscillating magnetic field perpendicular to the background field (called B1), the spins start to precess around the B0 axis with a frequency close to the Larmor frequency, which depends on the gyromagnetic ratio of the specific nuclei and the background magnetic field :

$$\gamma = \frac{f}{B_0\ (T)} \tag{1}$$

where γ, f and B_0 are the gyromagnetic ratio(Hz/T), the Larmor frequency (Hz), and the background magnetic field (T), respectively.

This precession exhibits a decreasing amplitude of latitude and is characterized as a decaying oscillating signal. This decaying oscillating signal read by an RF coil is called a free induction decay (FID) signal[1]. As stated in Equation (1), the resulting frequency is dependent on the magnitude of the background field (B0), so any change in the B0 will affect the frequency of the FID signal, which later can be translated to the change in magnetic field:

$$f = \gamma B \rightarrow (f_0 + \delta f) = \gamma(B_0 + \delta B) \rightarrow \ \delta B = \gamma^{-1}\delta f \ \ where \ \ \delta f = \frac{\Delta\phi}{\Delta t}. \tag{2}$$

Deionized (DI) water enclosed in a glass capillary was selected as the base structure for generating NMR signals, preventing evaporation over long periods and serving as a solid base for RF coil fabrication. This choice ensures that the free induction decay (FID) frequency is compatible with the RF equipment in commercial scanners, allowing for consistent and repeatable results. To minimise the nonlinear magnetic effects of a dipole field across large volumes, the amount of water used was kept as small as possible. At the same time, ensuring the scanner's analogue-to-digital converter (ADC) could detect the FID signal's amplitude within a reasonable acquisition time. Finally, an RF transceiver composed of a multi-turn pickup coil and a tuning and matching circuit was constructed around the glass tube to excite the spins and detect the NMR signal in the form of FID.

2. Soft Magnetic Materials at High Field

At high magnetic fields, the magnetization of ferromagnetic objects is saturated and aligns with the direction of the magnetic field. The saturation magnetization of soft magnetic materials, such as iron, could be greater than the saturation magnetization of hard magnetic materials, such as neodymium magnets. We performed magnetic hysteresis measurement using a vibrating sample magnetometer to show the difference in saturation magnetic field (Supplementary Figure 3).

The direction of the magnetic field is calculated using magnetic energy minimization [2]:

$$E = \min_{\phi} \frac{1}{2}\, N m_s^2 \, sin^2(\phi - \theta) - m_s H cos(\phi), \tag{3}$$

where $N = n_r - n_a$, and n_r and n_a are the demagnetization factors in axial and radial directions, and $2n_r + n_a = 1$. We calculated the demagnetization values according to Beleggia M. et al[3] for different aspect ratios of the tracker, a = L/D (Supplementary Figure 4b). We calculated the N at different aspect ratios (Supplementary Figure 4c). Later, we calculated the magnetization angle of an iron tracker for different shell thicknesses and a = 3 (Supplementary Figure 4d). For the solid cylinder, the magnetization angle reaches the maximum of ϕ=2.9° at θ=45° tracker angle and increases up to ϕ=4° as the wall thickness of the tracker increases. Next, we calculated the magnetic field created by a 1 emu magnetic tracker whose magnetization is rotated by different angles up to 4° with respect to the B_0 field.

3. Dipole magnetic field in NMR magnetic sensing

The magnetic field created by a magnetic dipole is calculated as follows

$$\mathbf{B}_{\mathrm{d}}(\mathbf{r}) = \frac{\mu_0}{4\pi} \left(\frac{3\mathbf{r}(\mathbf{m}\cdot\mathbf{r})}{r^5} - \frac{\mathbf{m}}{r^3}\right), \tag{4}$$

where $\mathbf{r} = [\boldsymbol{x}, \boldsymbol{y}, \boldsymbol{z}]^{\mathbf{T}}$ and $r = \|\mathbf{r}\|$ are the position and distance from the dipole; $\mathbf{m}$ is the magnetic moment vector of the dipole. Due to the high static, $\mathbf{B_0} = B_0\,\hat{z}$, the magnetic field of MRI scanners, the magnetic moment could be approximated as $\boldsymbol{m} = M\,\hat{z}$ For soft magnetic dipoles, M is the saturation magnetic moment of the dipole. During NMR magnetic sensing, we measure the magnetic field contribution of inhomogeneities in the $\mathbf{B}_0$, including the magnetic dipole. The magnetic field at a point in space in the MRI scanner could be written as

$$\mathbf{B}(\mathbf{r}) = B_0\,\hat{z} + \boldsymbol{\Delta}\mathbf{B} + \mathbf{B}_\mathrm{d}, \tag{5}$$

where $\boldsymbol{\Delta}\mathbf{B}$ is the background inhomogeneities. Then, the net magnetic field difference on the NMR magnetic sensor, δB , could be written as follows,

$$\delta B = \sqrt{(\boldsymbol{B_0} + \boldsymbol{\Delta B} + \boldsymbol{B})^{\mathrm{T}}(\boldsymbol{B_0} + \boldsymbol{\Delta B} + \boldsymbol{B})} - B_0. \tag{6}$$

We could expand this equation with $\boldsymbol{\Delta}\mathbf{B} = [\Delta B_x, \Delta B_y, \Delta B_z]$ and $\mathbf{B}_\mathrm{d} = [B_x, B_y, B_z]$, as follows:

$$\delta B = (B_0^2 + 2B_0\Delta B_z + 2B_0 Bz + \Delta B_x^2 + \Delta B_y^2 + \Delta B_z^2$$

$$+2\Delta B_x B_x + 2\Delta B_y B_y + 2\Delta B_z B_z + B_x^2 + B_y^2 + B_z^2)^2 - B_0, \tag{7}$$

In general, $\mathbf{B}_0$ in MRI scanners is in the order of Tesla and above, while inhomogeneities and magnetic dipole fields are in the order of µT, except in very close locations to the magnetic dipole. Therefore, we could write,

$$\delta B = B_0\left(1 + \frac{2\Delta B_z}{B_0} + \frac{2Bz}{B_0} + \mathrm{O}(\cdot)\right)^{1/2} - B_0, \tag{8}$$

where $\mathrm{O}(\cdot)$ are the higher-order terms. Then, using $\lim_{x\to 0}\sqrt{1+2x} = 1 + x$, we observe that the magnetic field change on the NMR sensor is only in the *z* direction, i.e., in the $\mathbf{B}_0$ direction,

$$\delta B \approx \Delta B_z + B_z. \tag{9}$$

Therefore, by considering $\hat{z}$ as a unit vector in $\hat{\mathbf{z}}$ direction, we could reduce the magnetic field of the dipole to :

$$\mathrm{B}_\mathrm{d}(\mathbf{r}) = \frac{\mu_0\, M}{4\pi}\,\frac{3\|\hat{z}\cdot\mathbf{r}\|^2 - \|\mathbf{r}\|^2}{\|\mathbf{r}\|^5} \tag{10}$$

4. Magnetic field variation in the sensor

The FID signal is composed of NMR signals of distinct frequencies (f_i) in the capillary tube. We used the slope of the phase of the signal S to calculate the average magnetic field. As the magnetic dipole gets closer to the sensor, the magnetic field variation throughout the sensor head will increase. To simulate the effect of frequency distribution, we calculated the f_i frequencies using the finite element method with a 25 µm voxel (Supplementary Figure 6a-b) and calculated the mean frequency ($\bar{f}$) of all the voxels. Then, we calculate the magnetic field deviation,

$$B_{dev} = \gamma^{-1}(f_c - \bar{f}) , \quad (11)$$

where f_c is the frequency at the center of the sensor. Thus, the average magnetic field will be calculated with additional error compared to the calibrated position (Supplementary Figure 6c-e), limiting the closest distance we can get to the sensor.

5. Meissner effect

The Meissner effect refers to superconductive materials' ability to expel external magnetic fields by creating a persistent surface eddy current due to zero resistance. This expulsion effect has been used in superconductive "active" shielding to cancel external magnetic fields[4]. The shielding effect could also be observed in a cylindrical superconductor, such as an MRI scanner. To verify the effect of this shielding, we performed a magnetic field measurement experiment with a large Neodymium magnet with 1 cm^3 and a magnetic moment of 1200 emu using the NMR magnetic sensors. We connected the magnet to a 1m-long carbon fiber rod to eliminate our bodies' diamagnetic effect. Then, we move the magnet at the entrance of the MRI scanner's bore against the MRI's magnetic pulling force, meaning that the magnetic moment is in the same direction as $\mathbf{B}_0$ (Supplementary Figure 8a). We observed that the magnetic approaches to -550 nT, in a negative direction – at 75 cm, a 1200 emu magnet generates 568 nT in a positive direction – as the magnet approaches the MRI bore, and the field increases as we move away from the scanner (Supplementary Figure 8b).

Furthermore, we moved the magnet further in the MRI while recording the middle sensor of the hexagonal array, and the reference sensor was placed 8 cm away. We observed that the field direction was inverted as the magnet passed beyond ~50 cm, where the shielding coil was located (Supplementary Figure 8c). More interestingly, the further-away reference sensor measures larger negative magnetic fields compared to the middle sensor. This behavior could be explained by two shielding coils symmetrically placed on two sides of the MRI scanner. While the closest coil rejects the external magnetic field created by the large magnet, it creates a current in the second coil placed at the other end of the MRI scanner. This current caused a magnetic field equivalent but reversed in direction at the center of the MRI scanner. And since the reference sensor is closer to the second shielding coil, we observe a larger negative magnetic field value. These experiments showed that the NMR magnetic sensors also capture the magnetic field generated by the interaction of the permanent magnet and the MRI scanner's superconducting coils.

To better understand the effect, we performed finite element simulations in Comsol. We assume that the superconductive coil of the MRI scanner is a smooth cylinder. Then, the magnetic dipole field is squeezed in the cylinder, as shown in Supplementary Figure 7a-b, since the superconductor expels the magnetic field. The effect of the magnetic dipole decreases as we move further away from the magnetic particle. To simulate the magnetic field repulsion, we used magnetic isolation boundary conditions in Comsol, and we swept the different cylinder diameters for a 1 emu magnet placed at the axis of the MRI scanner. We observed that as the radius of the cylinder decreases, its shielding effect on the dipole field increases (Supplementary Figure 7). Later, we focused on the 30 cm diameter of our small animal MRI scanners' superconductor coil. We simulated the magnetic field by moving the magnet in the radial direction. We observed an asymmetry in the magnetic dipole field as the magnet approached the coil.

These simulation results illustrate the potential effect of the Meissner effect on the dipole magnetic field and explain the reduction in tracking accuracy as the tracker moves away from the sensor array in our small animal MRI scanner. Although the effect would be much smaller in a larger-bore clinical human MRI scanner, a more Meissner effect-aware dipole modeling would be necessary to increase the tracking accuracy in the larger workspace. However, it is important to note that our simulation assumes a continuous superconducting surface. The exact position and geometry of the main field coils are proprietary information of the MRI producers and are not available for modeling. Therefore, such dipole modeling is not included in this work.

6. Workspace scaling

While the presented system has been limited to an NMR magnetic sensor array of seven with a fixed sensor distance, it is possible to increase the tracking precision and extend the workspace by changing the distance between the sensors and adding more sensors to the system. To demonstrate the relationship between sensor separation d_s and tracking precision, we calculated the tracking precision for a hexagonally placed sensor array for a 1 emu tracker at different distances on the center line of the array (Supplementary Figure 11a-b). We observed that the tracking precision is inversely proportional to ($1/ d_s$) at large distances, while there is an optimal sensor separation at small distances (Supplementary Figure 11c). The optimal sensor separation for tracking between 50-75 mm is between 20-30 mm, which aligns with the distance used in the presented NMR sensor array. In theory, we could increase the workspace by distributing the sensors to a larger area.

However, increasing sensor distance has a diminishing return in terms of precision, and we also decrease the precision in close-by points as separation increases. As a practical note, sensor separation is also limited by the MRI scanner's bore radius.

Another alternative to increase the workspace is to increase the number of sensors in the array. To demonstrate the concept, we repeated the analysis for different sensor numbers, 3, 7, and 19, while fixing the sensor separation to 20 mm (Supplementary Figure 11d). We observed that the tracking precision increased with the increasing number of sensors (Supplementary Figure 11e). For instance, we could increase the workspace by 2 cm by increasing the sensor number from 7 to 19 (Supplementary Figure 11f). We can achieve a significantly larger workspace by distributing additional sensors in 3D space. In our study, we illustrated various 3D sensor array configurations in different medical intervention scenarios, focusing on three specific regions of the body while utilizing the experimental noise floor and the mentioned simulation techniques. First, we examined endonasal routes to the skull base using an array of 19 sensors arranged in a triangular format across three rings to cover the average human head. One ring is positioned at the top of the head, while the other two rings encircle the forehead and nasal areas. As shown in Supplementary Figure 12a-c, the 2D precision map indicates that most brain areas have a precision of more than half a millimetre, and there is over 1 mm of precision from the nasal area to the brain at a depth of up to 3 cm. As illustrated in Supplementary Movie 2, the brain region maintains the half-millimeter precision throughout the entire brain volume.

Next, we analyzed transfemoral access via the common femoral artery, located about 2 cm below the inguinal ligament. For this analysis, we used the same six-sensor ring array around the thigh, demonstrating the extensive workspace available (Supplementary Figure 12d-f). The precision map indicates that we can achieve better than 1 mm accuracy up to 7 cm deep into the tissue until we reach the bone. By cascading the rings, we can cover a much larger volume if necessary; in this case, we utilized a total of 24 sensors arranged in four arrays.

Finally, we investigated the abdominal area, the largest region. Numerous procedures may require access to internal structures, such as suprapubic cystostomy, which targets the anterior bladder wall; peritoneal dialysis (PD) catheter insertion into the peritoneal cavity; or percutaneous nephrostomy, which provides access to the kidneys from the posterior flank. To ensure access to the entire abdominal volume, we repeated the triangular configuration three times in succession,

creating an array of 39 sensors, which formed a belt around one side of the abdomen (either posterior or anterior access). As evident in the 2D precision slice taken 7 cm deep in the body (Supplementary Figure 12g-i), we achieved a precision of over 1 mm across the entire 40 cm width of an average body. By cascading this pattern, we can effectively cover the whole abdominal area, rather than focusing on just one specific procedure. All the analyzed areas are represented in 3D space, with the results showcased in Supplementary Movie 2.

7. Flexible tracker shape effect

We developed a finite element simulation to explore the effect of shape change on the position estimation of flexible soft magnetic trackers. Assuming a 1D rod model with equally distributed magnetization, we divided the 20 mm flexible tracker into 500 μm segments. We calculated each segment's magnetic field contribution on the sensors using small dipoles. We observed the estimated position by the particle filter at different tracker bending angles between 0° and 90° in horizontal and vertical states over time (Supplementary Figure 19a-d). A maximum absolute error of 2.4mm and 1.5mm was observed for vertical and horizontal cases, respectively, compared to the simulated center of the model (Supplementary Figure 19c-f)

8. Balloon volume effect

Another finite element simulation was developed to investigate the volume change in a stationary cylindrical balloon. The cylinder was divided into voxels ranging from 5 μm^3 to 100 μm^3, and the effect of each voxel, treated as a small dipole with equally distributed magnetization, was calculated on the sensor (Supplementary Figure 22). We observed the difference between the simulated center and the estimated position while changing the volume with a constant aspect ratio of 4.1. The position error is 5.5 mm at 2 ml volume and goes to zero as we get closer to a point dipole.

9. Tracking with intra-operational imaging

To validate tracking operation in the presence of biological tissue and imaging capability, we conducted an ex vivo experiment inside a porcine brain. A 3d printed stage was used to position the 4 cm diameter surface coil and the brain in the MRI bore, and a 2mm diameter Teflon tube was placed inside the brain to function as our channel. First, sagittal and oblique images were taken using 2D MR imaging (Localizer sequence, Bruker), followed by a sensor calibration and tracking

procedure Finally, the estimated position and images were overlaid using the MRI coordinate system to show the precision of tracking inside the porcine brain.

10. MRI sound comparison

We compared the acoustic noise of the NMR tracking sequence with standard 13 sec 2D MR imaging (Localizer sequence, Bruker), 1 Hz 2D GRE sequence, and 20 Hz 1D projection sequence (5) by recording each sequence at 75 cm distance from the MRI bore (Supplementary Figure 23a). We observed that the acoustic noise caused by gradient coils has reached over 70 dB in faster 2D imaging and 1D projection. Since NMR tracking does not require gradient hardware, it results in substantially lower noise – only the cryogenic pump noise is present in the background. We plotted the decibel levels compared to a sound level in an empty office as a reference (Supplementary Figure 23b).

11. Multi tracker concept

We conducted tracking experiments using two trackers to explore the possibility of a multi-tracker system with the current number of sensors. A stage featuring parallel channels (Supplementary Figure 10a) was used to move the trackers within the workspace (Supplementary Figure 24 and 25). The experiment was conducted at three different distances: 4cm, 2cm, and 1cm. In each experiment, one tracker was moved 1 cm using a Teflon guidewire, while the other remained stationary. To estimate the positions using the particle filter, we increased the number of estimated states in the algorithm from three to six to account for the second dipole. The particle filter can estimate the positions of multiple trackers simultaneously; however, the accuracy and precision of these estimates decrease compared to single-dipole experiments. When the trackers are spaced 20 mm and 40 mm apart, the particle filter can still estimate their positions within a workspace of 60 mm and 40 mm away from the sensor array (Supplementary Figure 24). In contrast, for single tracker scenarios, the workspace, with accuracy exceeding 2 mm, extends to 80 mm; however, as trackers are placed closer to each other, at 10 mm, the dipole field of two trackers becomes indistinguishable for the particle filter, leading to a further reduction in position estimation accuracy (Supplementary Figure 25). We believe that this problem could be addressed in future robotics systems by incorporating system dynamics into the prediction stage of a particle filter.

12. Magnetic artifact in MR images

Placing a magnetic tracker inside an MRI machine will disturb the magnetic field in the shape of a magnetic dipole in three-dimensional space. This disturbance is significant in a volume surrounding the tracker, leading to a substantial change in the NMR frequency of water, as stated in Equation (1). As a result, the volume will not be excited within the utilized RF signal bandwidth, causing complete signal loss in that area. At greater distances from the tracker, the disturbance in the magnetic field is not strong enough to significantly shift the frequency. However, it can still distort the image by affecting the calculations of the gradient fields, which can show itself as a skewed image. Two ex vivo experiments using porcine brain were conducted to demonstrate this effect in a real tissue. In the first experiment, the tracker was inserted inside the brain in 10 steps, and an MR image was taken after each step. The entire process is illustrated in Supplementary Figure 26 and summarized in Supplementary Movie 4. In the second experiment, a preoperative image was first taken. Then, NMR sensors tracked the tracker's position throughout the insertion process. Finally, a postoperative image was taken. The preoperative and postoperative images are overlaid with the estimated position of the tracker. They are shown in Supplementary Figure 27 as snapshots of the process, and the whole procedure is documented in Supplementary Movie 3.

13. RF shielding

Each sensor consists of a miniaturised RF coil, which allows it to excite the volume inside and around the coil. This can lead to unintended excitation of nearby sensors or tissues. To minimize potential interference from the surrounding environment, the sensor is placed inside an aluminium box serving as an RF shield. To demonstrate the efficacy of the RF shield, we placed two NMR sensors, a shielded and an unshielded sensor, on top of a porcine brain, as shown in Supplementary Figure 28a. We performed 2D MR imaging experiments using the Localizer sequence with an excitation power of 80 microwatts, which matches the power used in NMR magnetic sensing. We acquire two sets of slice images: one from a plane passing through the sensors, and one from a plane passing through the brain, as shown in Supplementary Figure 28a. We observed that due to the shielding on one of the sensors, the MR images captured from each RF coil did not receive any signal from the water of the other RF coil (Supplementary Figure 28b-c). From the second set of slice images, we observed that while the shielded sensor did not receive any signal from the brain tissue, leaving only imaging noise (Supplementary Figure 28d), the unshielded sensor received a

significant signal from the tissue (Supplementary Figure 28e). This experiment confirms the shield's effective role and shows no interference between sensors while shielded.

14. RF heating experiments

To demonstrate that the NMR magnetic tracking does not cause RF-induced heating problems, we performed controlled imaging and sensing experiments. We compared the temperature increase during the extended imaging sequence and sensing in two different sets of experiments. First, the experiments were conducted using 80 ml of 2.6 M saline solution in imaging and sensing mode. A hollow tracker was then placed inside the solution, and the experiments were repeated. A Gradient Echo (GRE) sequence with a repetition time of 10 ms, an RF pulse duration of 0.54 ms, and a power of 90 W was used for imaging mode, while the sensing mode remained unchanged. Each experiment was conducted over 20 minutes, and sufficient time was allowed for the system to stabilize between each experiment. Three temperature sensors were utilised during each experiment, two placed next to the tracker and one far away to measure the environment's temperature. During the imaging period, we observed a consistent temperature increase due to the high conductivity of the solution (see Supplementary Figure 29). However, we noted no significant change in temperature after placing the hollow tracker inside the solution. This is expected because the geometry of the tracker is much smaller than the signal wavelength, resulting in a practically negligible absorption rate. Additionally, no substantial temperature change was detected in the sensor mode, which reflects our expectation that no RF radiation exists outside the sensor shielded box.

15. Magnetic force and torque safety

The magnetic force and torque are, in general, important safety risks in MRI-compatible devices. We observe magnetic forces at the entrance of the MRI bore due to the high magnetic gradients, which could reach 20 T/m in 7 Tesla MRI scanners, and magnetic torques at the imaging center of the MRI scanner, where we have high uniform magnetic fields. We calculated the maximum magnetic force exerted on the tracker with a 1 emu magnetic moment using

$$F = \mu_0 V M_s \frac{\partial H_z}{\partial z}, \tag{12}$$

as 19 mN in our 7 T MRI scanner [6]. A human operator or a mechanical insertion mechanism could easily overcome this force. Moreover, the magnetic force on the magnetic tracker disappears once the tracker is at the imaging center. The MR imaging gradient would apply a small force on the tracker; however, due to the very low strength of these gradients, typically less than 60 mT/m, the magnetic force during imaging would be in the order of or less than a couple of μN, which would barely vibrate the tip of a guidewire. Since our NMR magnetic tracking does not utilize gradient coils, we do not need to consider imaging gradient-based forces either.

On the other hand, the magnetic field at the center of the MRI scanner applies a magnetic torque, aligning the tracker's magnetic easy axis with the field direction. Since we used soft magnetic materials rather than hard magnetic materials, this magnetic torque is much smaller than in our previous work. We calculated the maximum torque acting on the 1 emu tracker with a 0.6 mm diameter and a 2 mm height solid tracker, using[2]

$$\tau = \frac{\mu_0 V N M_s^2}{2}\,. \tag{13}$$

For the aspect ratio of 3.33, we calculated N from Supplementary Figure 4c. Then the maximum torque can be calculated as 0.3 mN/m, which is an order of magnitude smaller than the torques in our previous work. A flexible tracker with the same 1 emu has much lower torque since the magnetic materials are distributed over a larger volume with lower magnetic density. That being said, we should note that there is no literature on the calculation of magnetic torque on flexible magnetic materials composed of magnetic microparticles and an elastomer matrix at under-saturation conditions.

Tables

Supplementary Table 1. Comparison of wireless tracking systems. ***Statistics have not been reported explicitly. MT: magnetic tracking, MGT: magnetic gradient tracking, EMT: electromagnetic tracking.

	**Tracking Modality**	**Tracker Size (mm^2)**	**Temp. Res.**	**Penet. Depth**	**Tracking Acc.**
Son [5]	MT	12.8 × 6.4Ø	5 ms	5 cm	2.1 mm
Gleich[6]	EMT	1.9 × 0.8Ø	25 ms	30 cm	2 mm at 10 cm*
Sharma [7]	MGT	20 × 8Ø	300 ms	40 cm	2 mm at 12 cm*
Osaki [8]	EMT	15 × 4Ø	100 ms	15 cm	<1 mm at 15 cm
Arx [9]	MGT	6 × 3Ø	100 ms	10 cm	<1 mm at 10 cm
**This work**	MT	2 × 0.6 Ø	30 ms	8 cm	2 mm at 8 cm

Figures

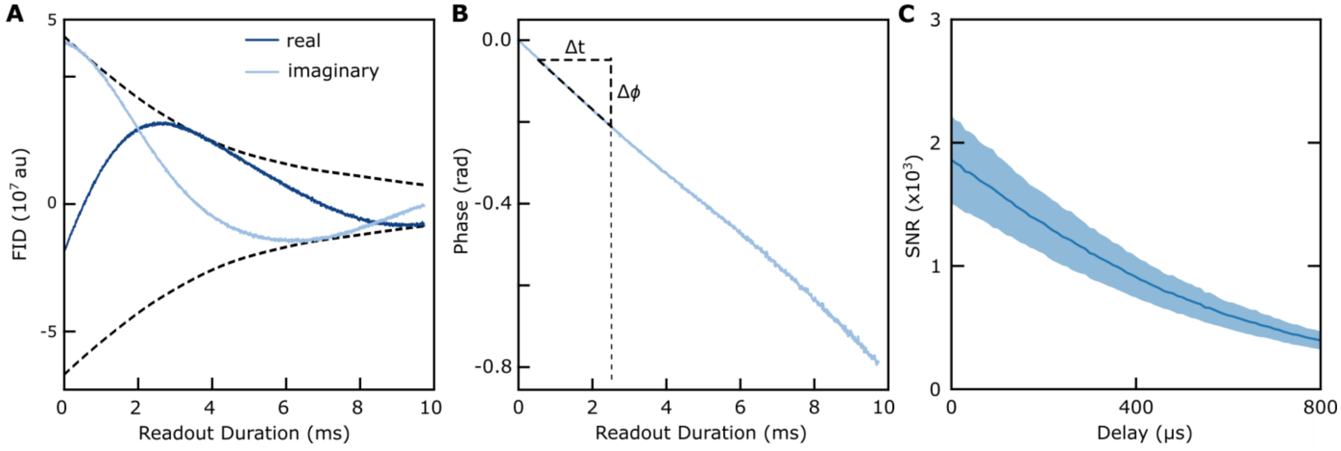

Supplementary Figure 1. FID signal measurement and processing. A) Real and imaginary parts of the generated FID signal from the scanner. B) Phase accumulation is calculated using an FID signal. A straight line is fitted to the last 2 ms of the linear region (2.5 ms) to measure the frequency. C) SNR of the signal as a function of acquisition time.

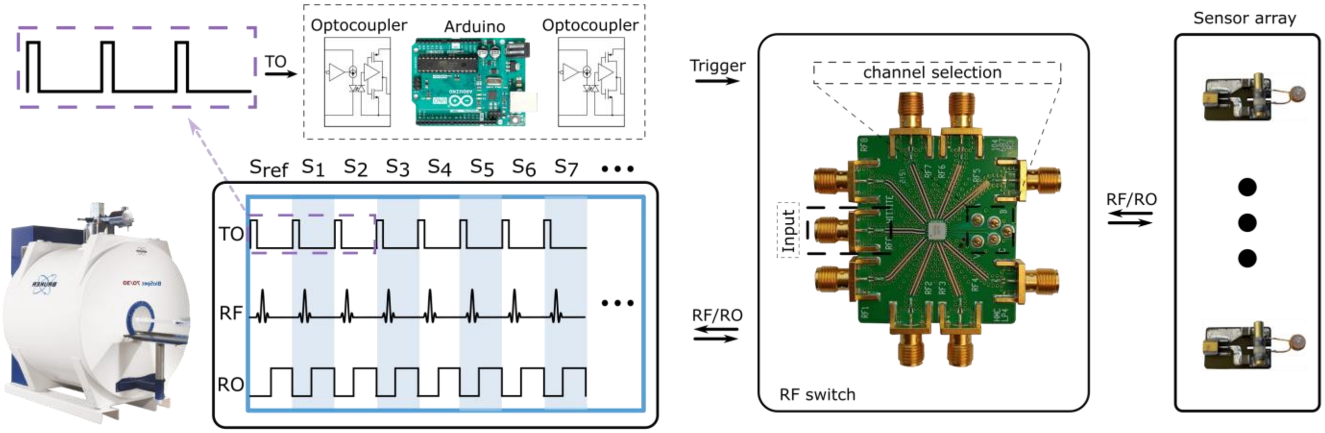

Supplementary Figure 2. Switching system used for a multi-sensor array. The MRI sequence consists of a 1 ms TO (Trigger Out) pulse for the switch trigger, followed by a 0.2 ms RF sinc pulse, and finally, 2.5 ms of RO (Read Out) for signal acquisition. Optocouplers decouple the Arduino from the MRI trigger signal and channel selection logic unit of the switch.

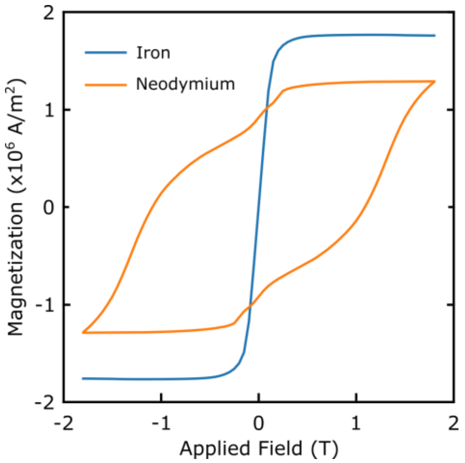

Supplementary Figure 3. Comparison of saturation magnetization between soft and hard magnets. The VSM magnetic hysteresis measurement of iron (blue) and neodymium (orange) magnets is shown.

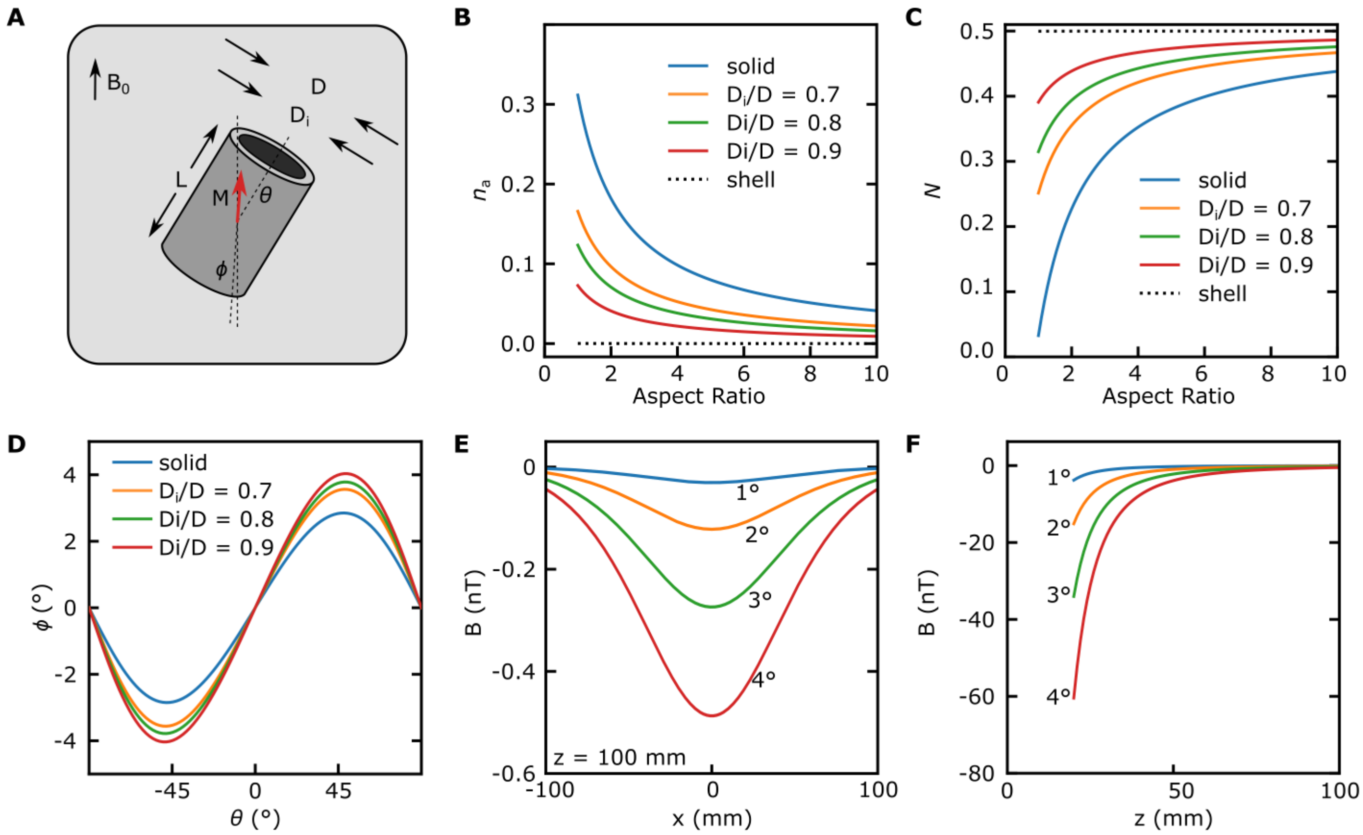

Supplementary Figure 4. Orientation-dependent tracking of the solid tracker. A) The schematic of a soft magnetic hollow cylinder. B) The axial demagnetization constants of the cylindrical shells at different aspect ratios. The demagnetization constants are calculated using the analytical model [3]. C) The difference between axial and radial demagnetization constants. D) The magnetization angle of soft magnetic iron trackers as a function of tracker angle with respect to the $\mathbf{B}_0$ field direction. E) The deviation from the magnetic field of the aligned magnetic dipole at 100 mm distance to the sensor as a function of radial distance. F) The deviation in the magnetic dipole field as a function of distance to the sensor.

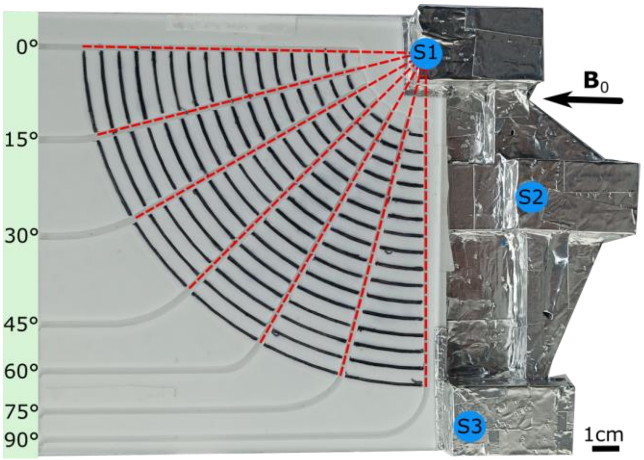

Supplementary Figure 5. Dipole measurement setup. The 3D-printed stage is used to measure the magnetic field of a 1 mm diameter steel bead. The dashed lines are the 10 cm distance from the sensor for each channel. Each channel has a specific angle compared to the B_0 field, which is shown on the left side. Sensors are shielded on the stage with aluminum tape.

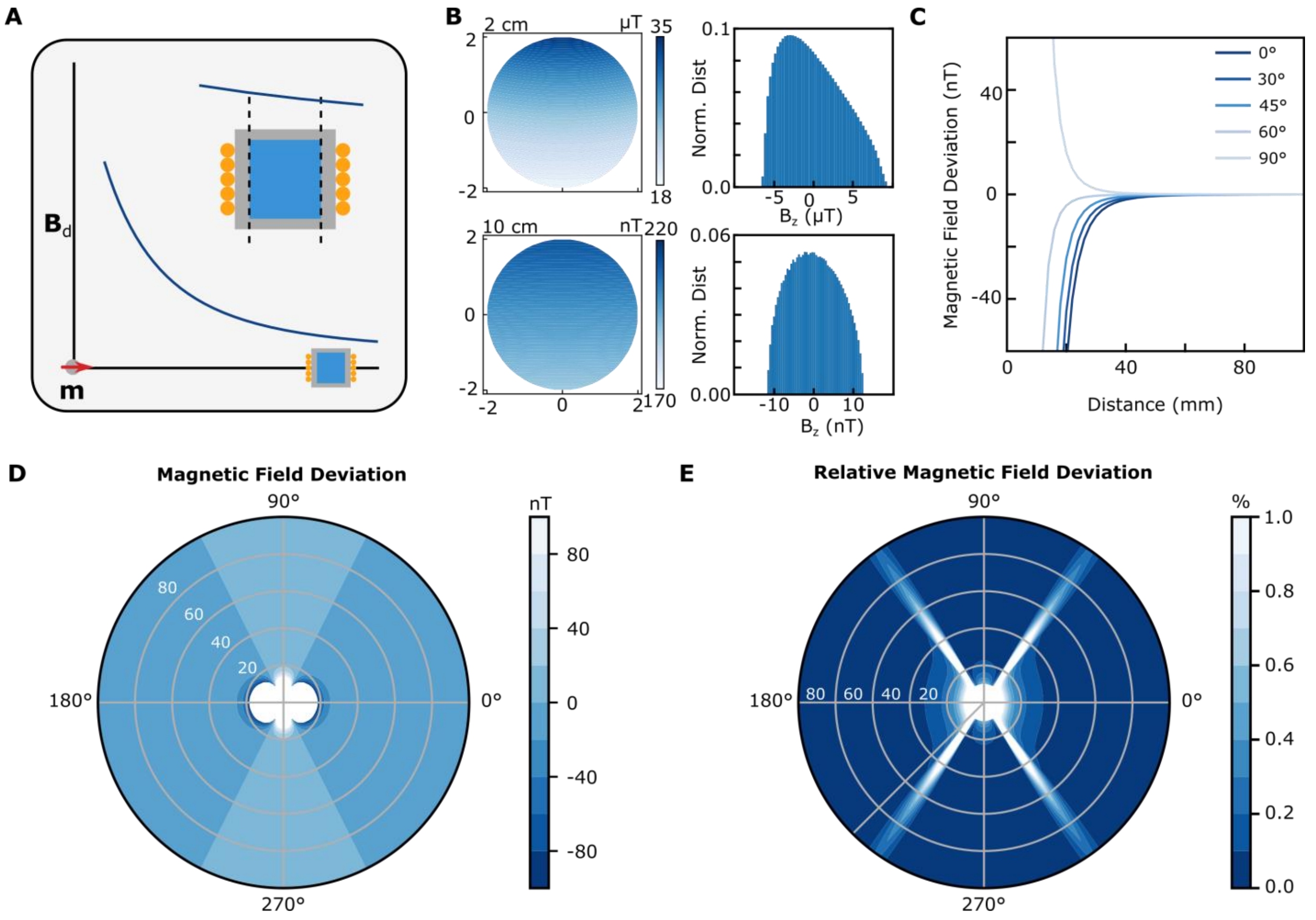

Supplementary Figure 6. Effect of sensor volume on measurements. A) Dipole field variation on the sensor in relation to distance. B) Magnetic field distribution on the sensor head at 2 cm and 10 cm distances from the dipole**.** C) Magnetic field deviation from a point sensor at different distances and angles compared to the sensor center. D-E) 2D map of field deviation (D) and relative field deviation (E) as a function of dipole position on the plane. Iso-centric circles indicate the distance from the sensor located at the center of the plane**.**

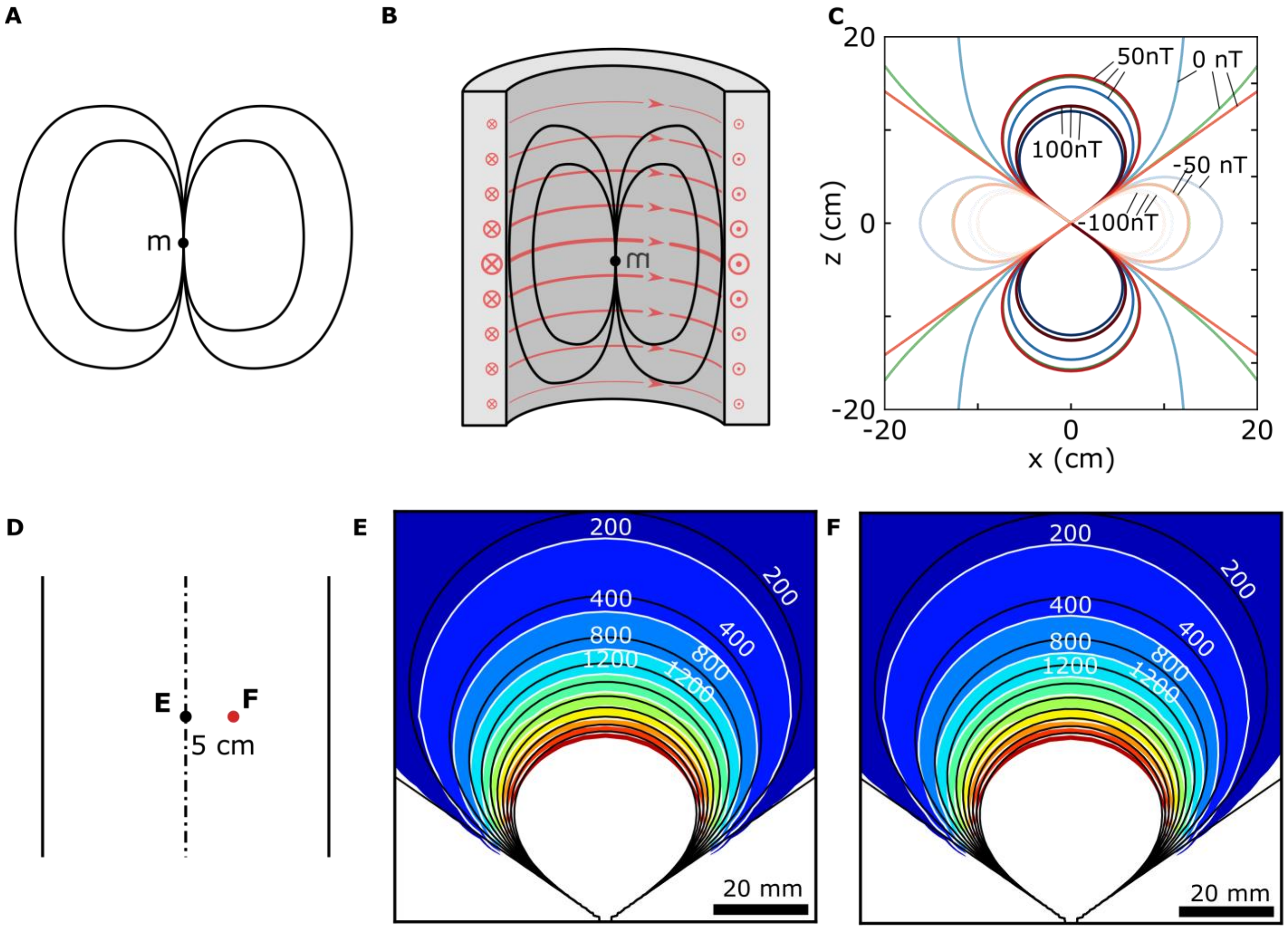

Supplementary Figure 7. Meissner effect simulation for a dipole inside an MRI. A) Magnetic field lines of a dipole in free space. B) Magnetic field lines of a dipole in the center of a superconductor cylinder. The magnetic material causes surface currents in the superconductor, which reduces the measured magnetic field. C) The simulated magnetic field of a 1 emu magnetic dipole in three different superconductor diameters. The red-colored map lines represent a dipole in free space. The green color map lines belong to the dipole in a 100 cm superconductor diameter, and the blue lines belong to the dipole in a 40 cm superconductor diameter. The different magnetic field values are shown in the plot. D) The off-centered magnet is 30 cm in diameter. E) The magnetic field of a centered magnet. The black lines are the dipole in free space. F) The magnetic field of a magnet shifted 5 cm to the right. The dipole field lost symmetry and leaned towards the left.

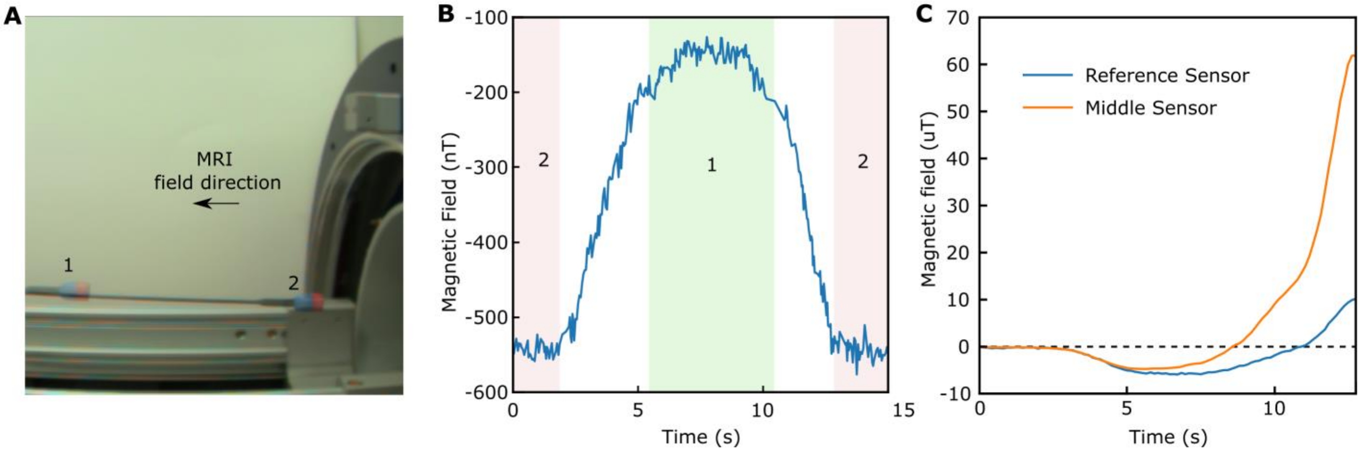

Supplementary Figure 8. External magnetic field effect. A) Movement of a large magnet outside of the MRI bore while measuring the magnetic field in the center of the MRI bore. B) Measured magnetic field outside of the MRI scanner. The green area is position 1, and the red area is position 2 in (A). The magnetic field decreases as the magnet approaches the MRI. C) Magnetic field measurement inside the MRI scanner. The magnet is moved in the MRI scanner beyond the shielding coils to show the inversion in measured field values. The blue data is the reference center placed 8 cm away from the center of the MRI scanner. Orange data is from the center of the MRI scanner.

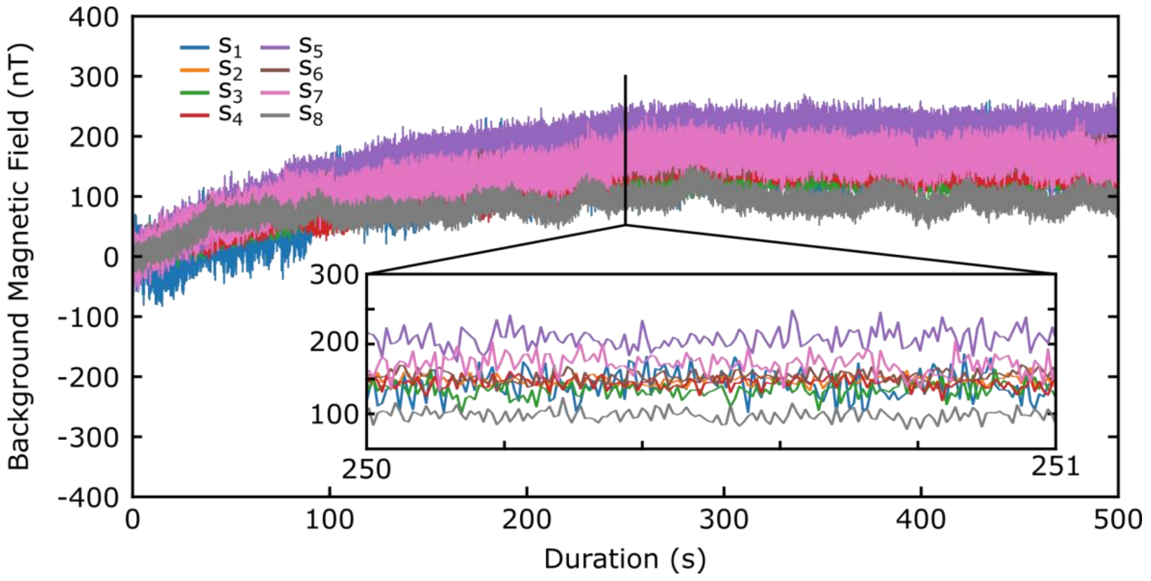

Supplementary Figure 9. Background measurement over time. The magnified plot illustrates the variation in the background field value for each sensor, indicating its dependence on position.

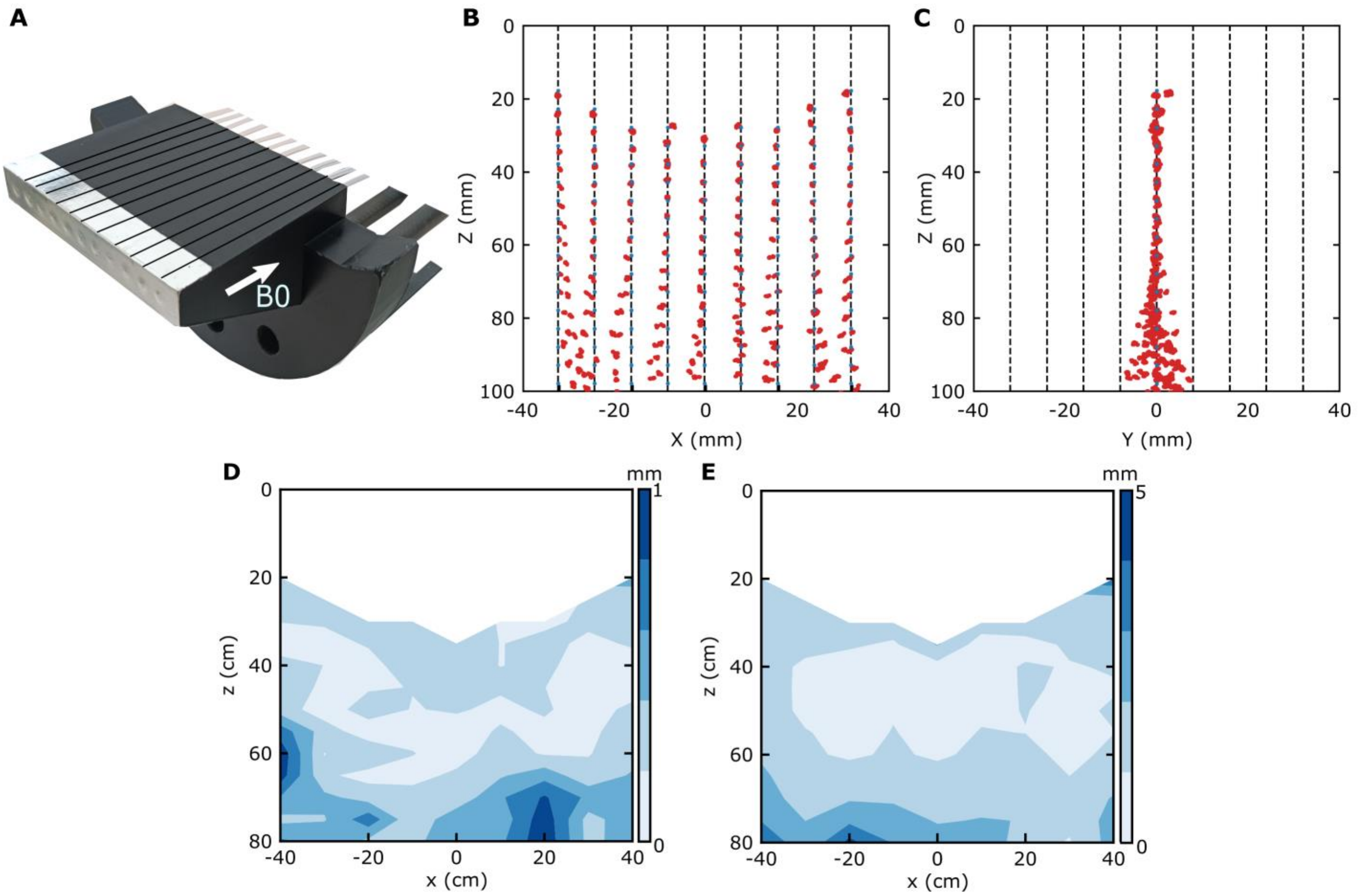

Supplementary Figure 10. Workspace measurement. Experiment. A) 3D printed stage with parallel channels. B-C) Real and estimated position of the tracker in Z-X plane (B) and Z-Y plane (C). The blue dots represent actual positions, and the red dots represent estimated positions. D) The precision (standard deviation) of the position error. E) The accuracy (mean) of the position error.

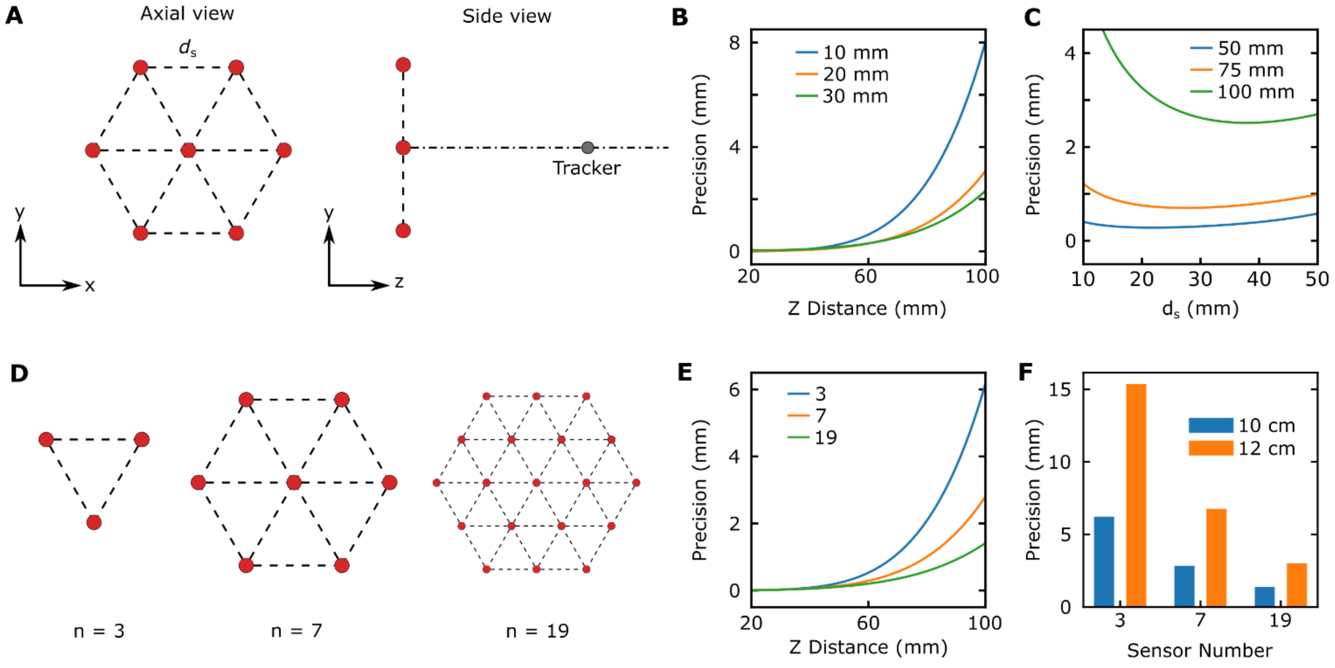

Supplementary Figure 11. Workspace scaling. A) Current sensor array arrangement. B-C) Estimation precision as a function of tracker distance (B) and space between sensors (C). D) Sensor number scaling. E-F) Comparison of estimation precision with different sensor numbers as a function of distance from the tracker.

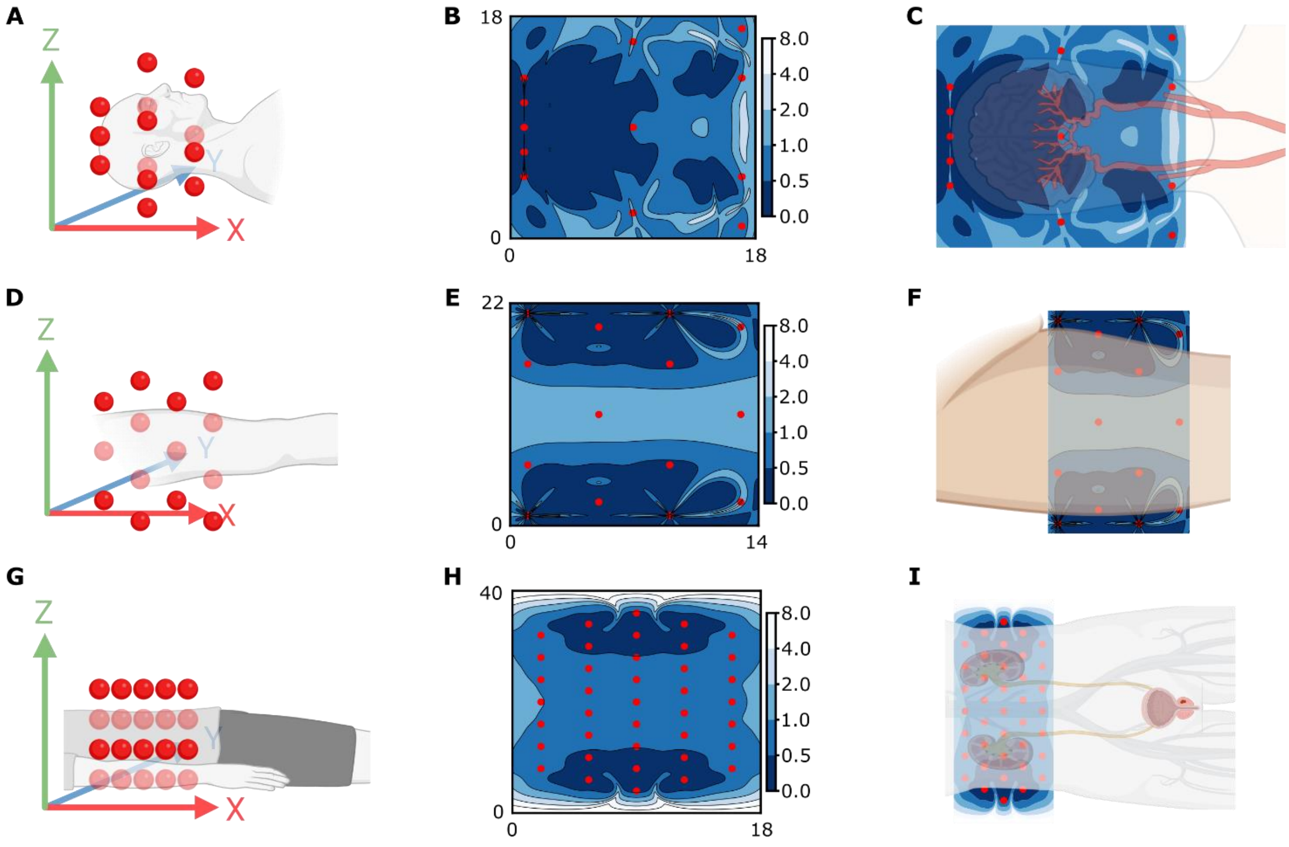

Supplementary Figure 12. Various 3D sensor arrays for medical interventions. A-C) A 3D array proposed for the head, with its precision map separate (B) and overlaid (C) on the head. D-F) A 3D array proposed for the thigh region and its precision map, separate (E) and overlayed (F) on the thigh. G-I) A 3D array proposed for the abdominal region and its precision map, separate (H) and overlayed (I) on the abdominal region.

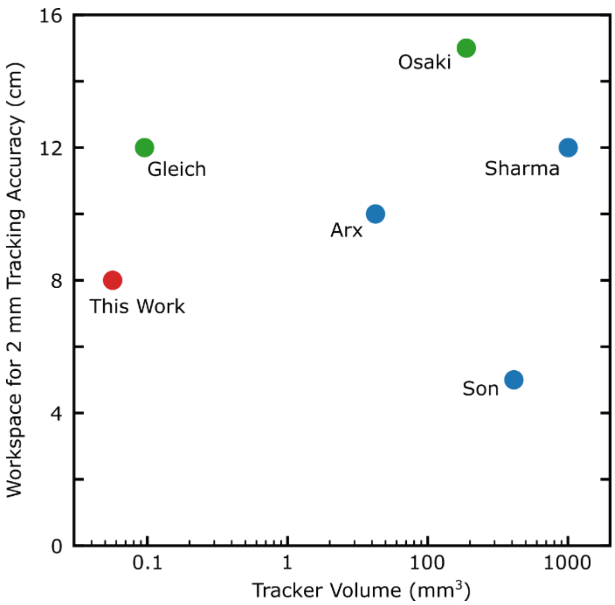

Supplementary Figure 13 Miniaturization comparison. It can be seen that in this work, we can track smaller volumes of magnetic material with better accuracy in a similar workspace [9-13]. The lower limit of tracking accuracy is considered to be 2mm. Blue dots represent magnetic tracking methods, while green dots represent EM tracking methods.

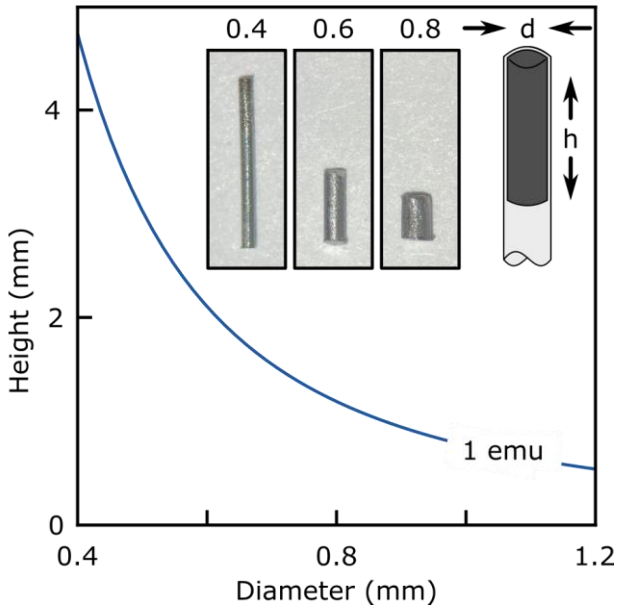

Supplementary Figure 14. Tracker miniaturization. Three 1 emu spring steel trackers with different diameters are shown.

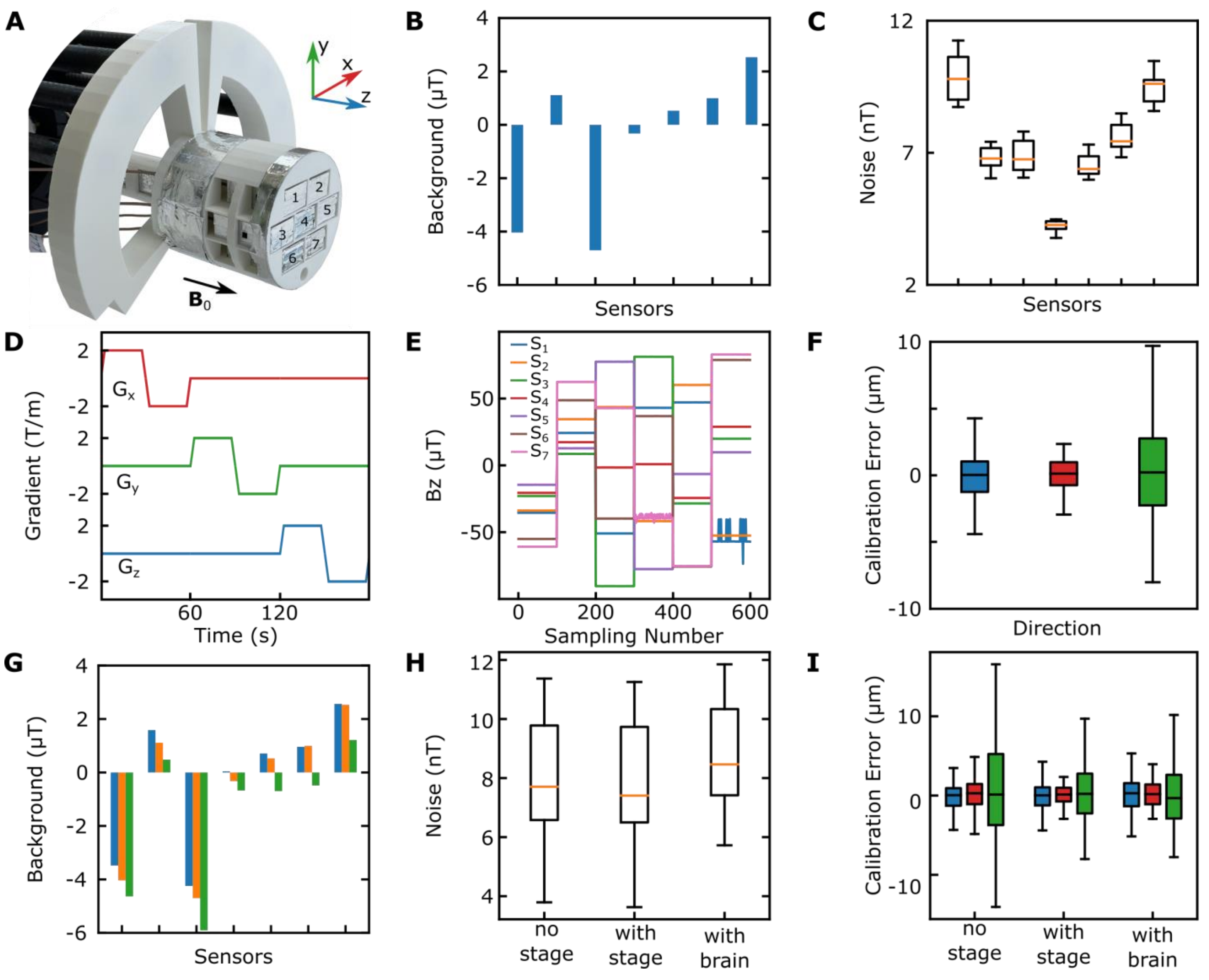

Supplementary Figure 15. Sensor calibration process. A) 3D printed stage for sensor array fixture. B) background measurement data. C) Noise measurement data. D) Gradient activation for position encoding of the sensors. E) Sensors read out during the gradient activation. F) Position error in 3d space, G-I) Comparing background field (G), background noise (H), and 3D position error (I) in 3 different states of no patient's stage, with stage, and having the porcine brain on the stage. Box plots display the median (center line), interquartile range (IQR, 25th–75th percentiles) as the box, and the minimum and maximum values as whiskers

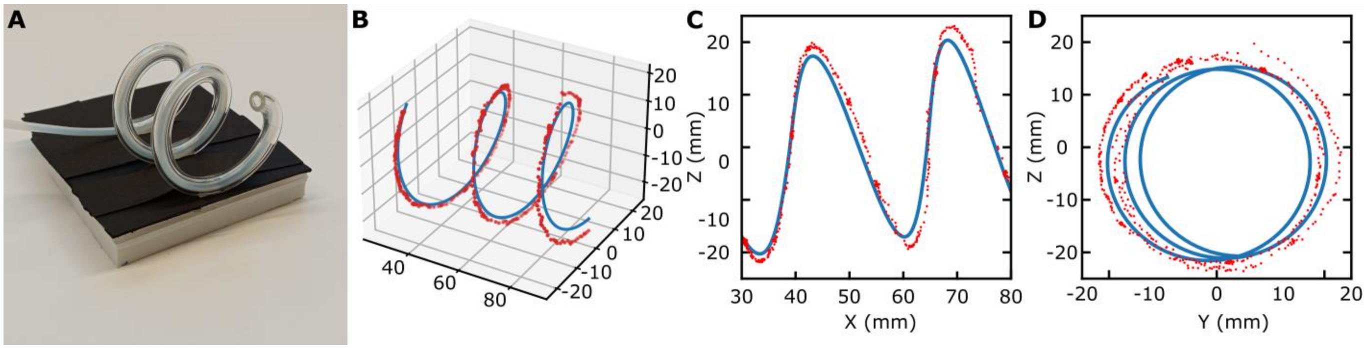

Supplementary Figure 16. Laser catheter experiment. A) The spiral channel is made out of glass. B-D) The real and estimated path taken by the hollow tracker in 3D space (B), Z-X plane (C), Z-Y plane (D). The solid line is the ground truth extracted from the spiral channel.

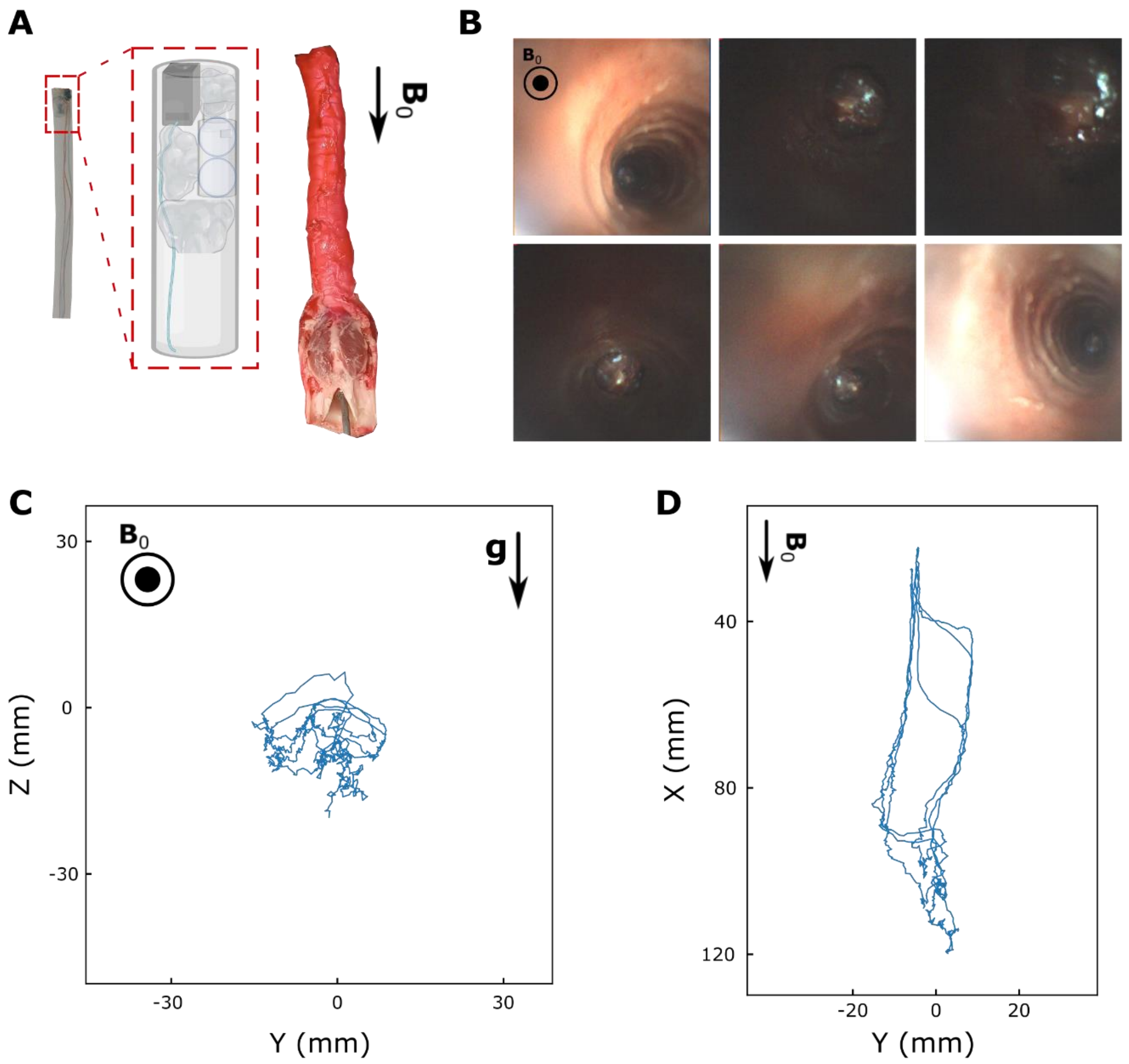

Supplementary Figure 17. Endoscope camera tip tracking inside the porcine oesophagus. A) Custom made endoscope camera system and the Porcine oesophagus used for the operation. B) endoscope camera feed inside the oesophagus. C) Axial view of position tracking throughout the whole operation. D) Coronal view of position tracking throughout the whole operation.

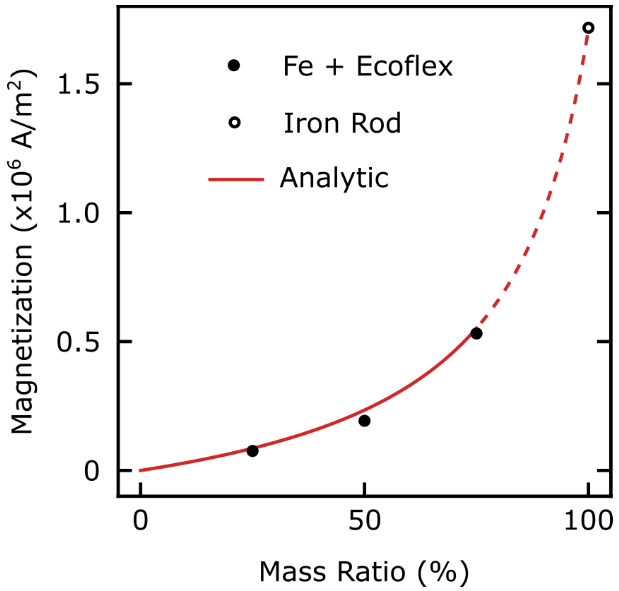

Supplementary Figure 18. The magnetization of the flexible tracker as a function of mass ratio. Solid dots are saturation magnetization measurements of different iron ecoflex mixtures. The circle is the saturation magnetization of solid iron. The red line is the analytic model for saturation magnetization. The above 75% analytic model is shown with the dashed line since EcoFlex does not cure properly at a higher mass ratio.

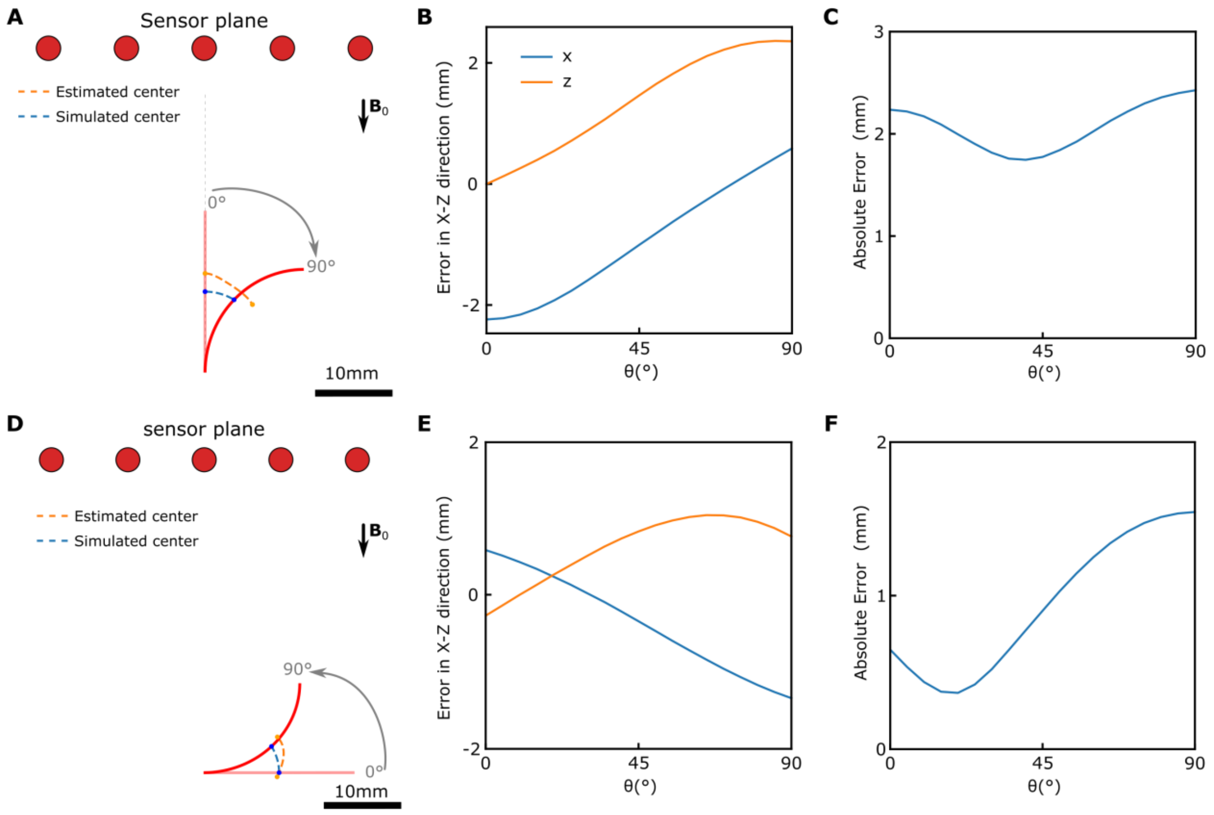

Supplementary Figure 19. Flexible tracker shape effect. A) Simulation environment for the vertical rod model. B-C) estimation error in X-Z direction (B) and absolute term (C). D) Simulation environment for horizontal rod model. E-F) Estimation error in X-Z direction (E) and absolute term (F).

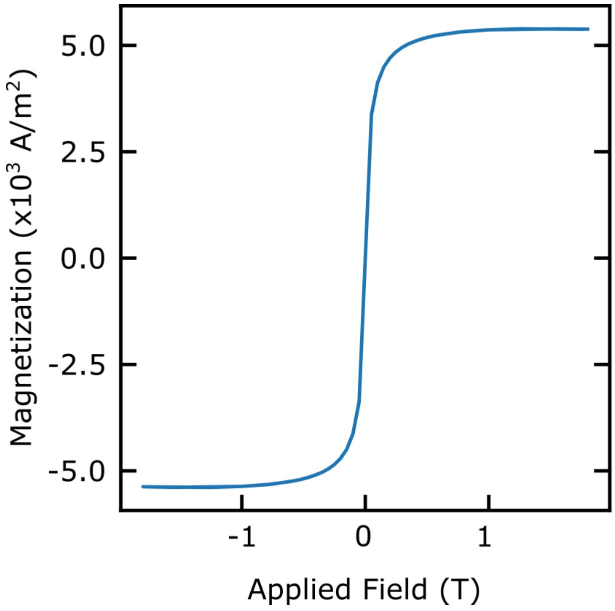

Supplementary Figure 20. The magnetization of iron-oxide ferrofluid. 10 uL of iron oxide nanoparticle solution with a 25mg/ml concentration was prepared for VSM measurement.

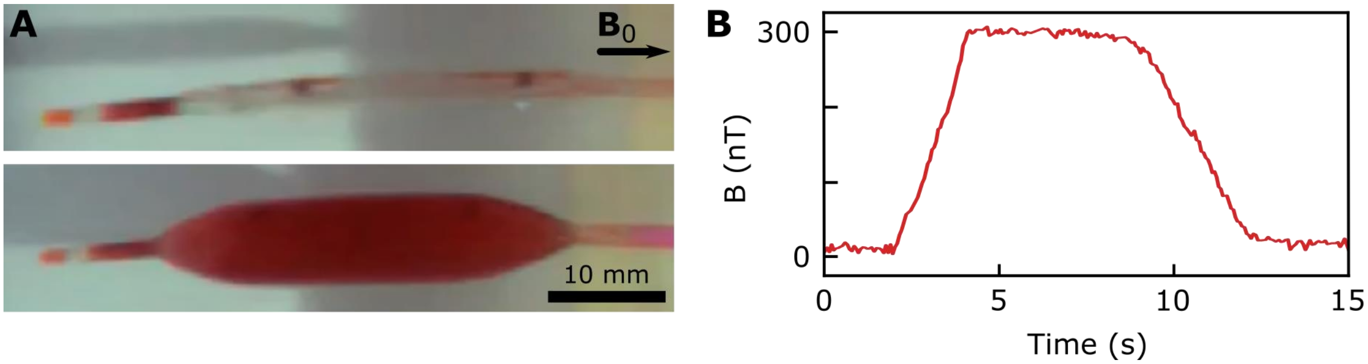

Supplementary Figure 21. Ballon catheter inflation-deflation. A) The inflation and deflation of a balloon catheter with an iron-oxide nanoparticle solution. B) The magnetic field measurement during the inflation of the balloon.

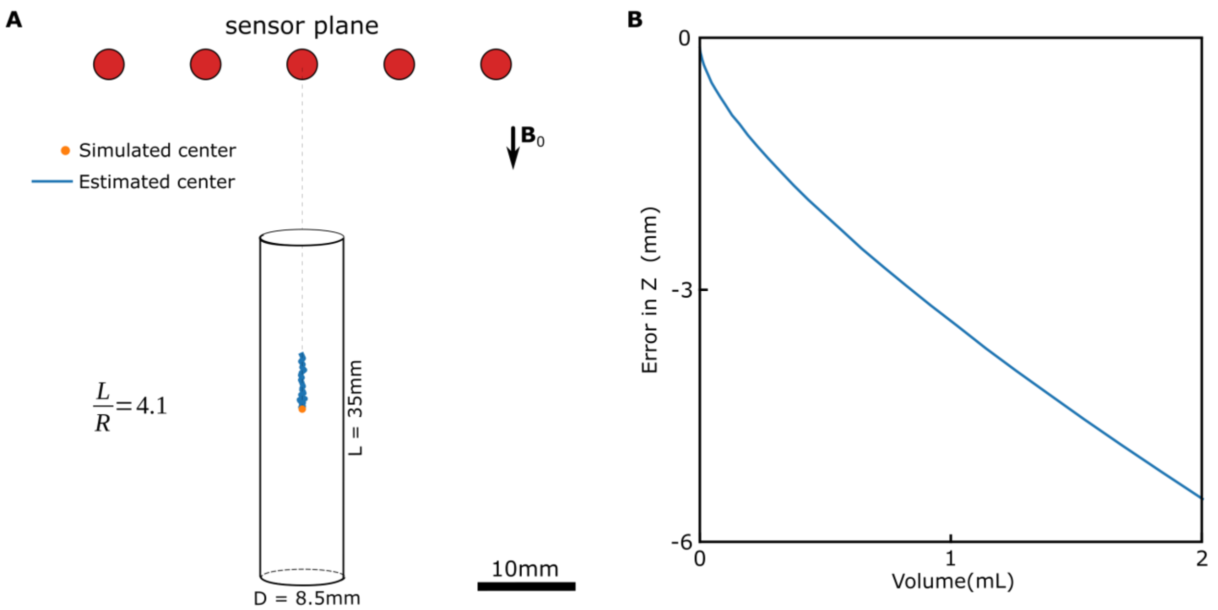

Supplementary Figure 22. Ballon catheter shape effect. A) Simulation Environment for the cylindrical model. B) Estimation error in Z direction

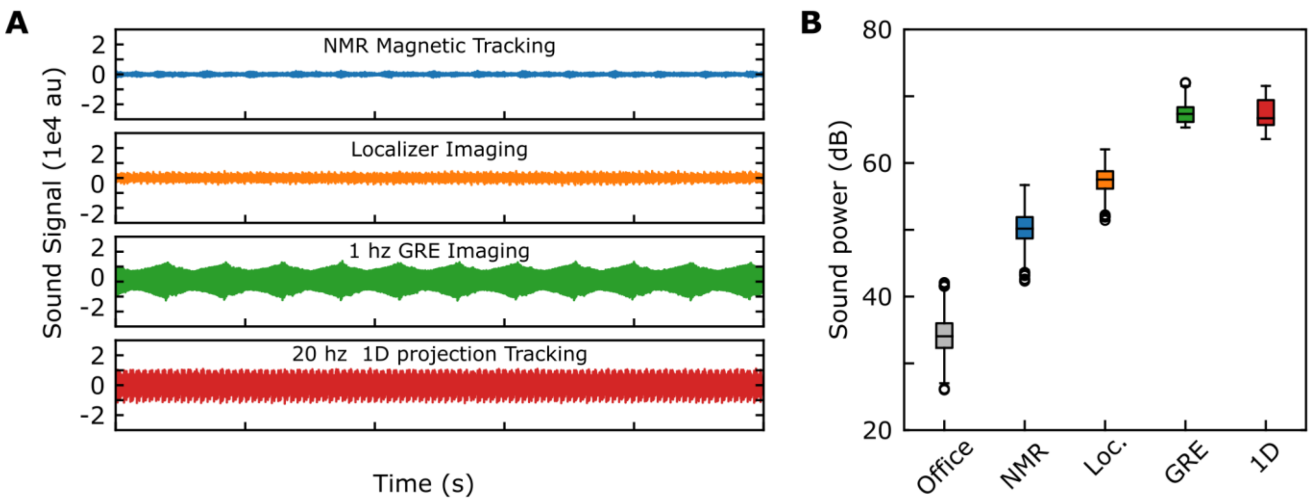

Supplementary Figure 23. Sound data comparison. A) Sound data of MRI in different MRI and NMR sequences. B) Comparison of signal power between different sequences. Box plots display the median (center line), interquartile range (IQR, 25th–75th percentiles) as the box, and the minimum and maximum values as whiskers

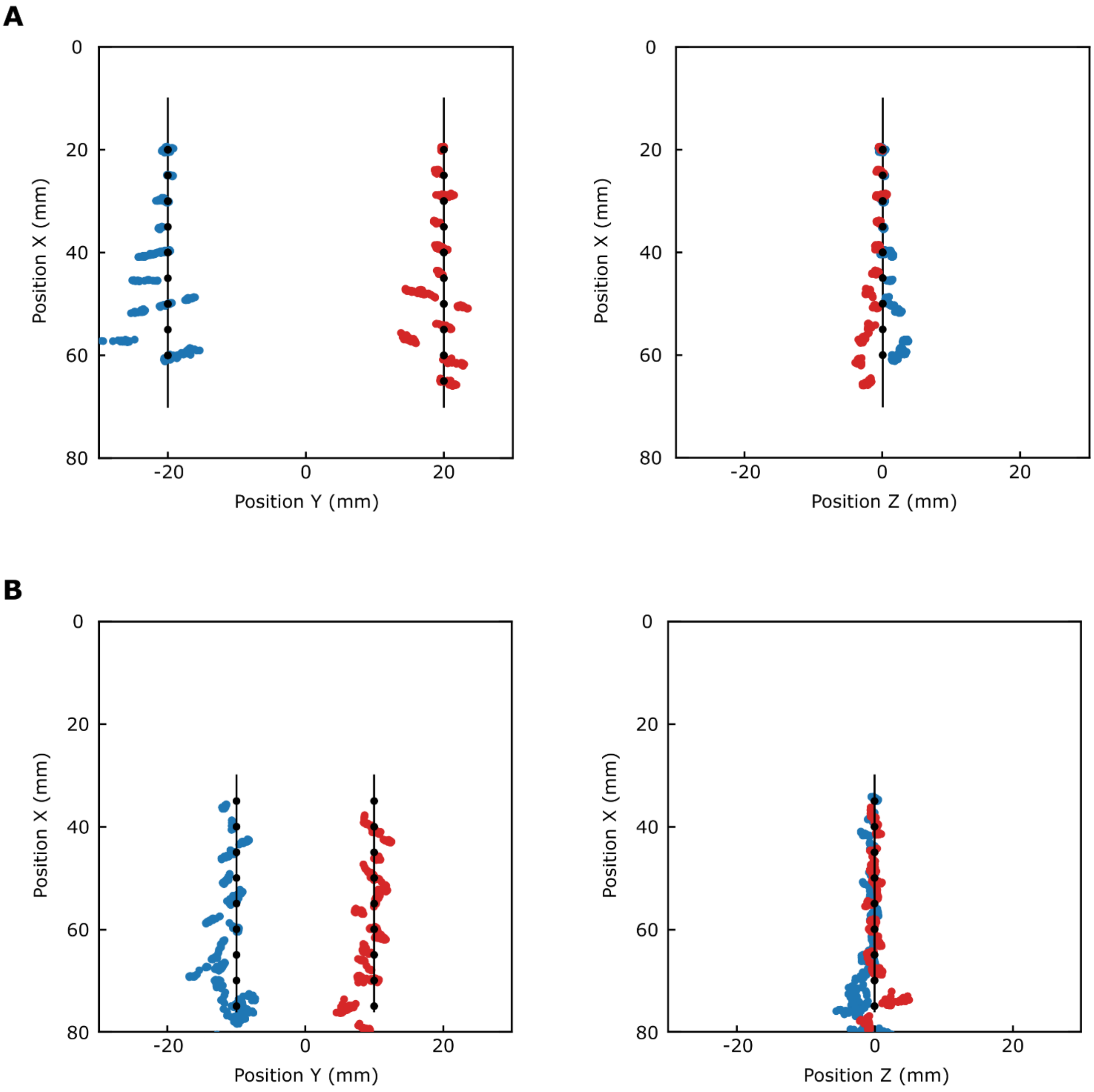

Supplementary Figure 24. Multi-bead experiment with a large distance between trackers. The position estimation accuracy of two trackers at a large proximity to each other, in 4 cm (A) and 2 cm (B), is investigated.

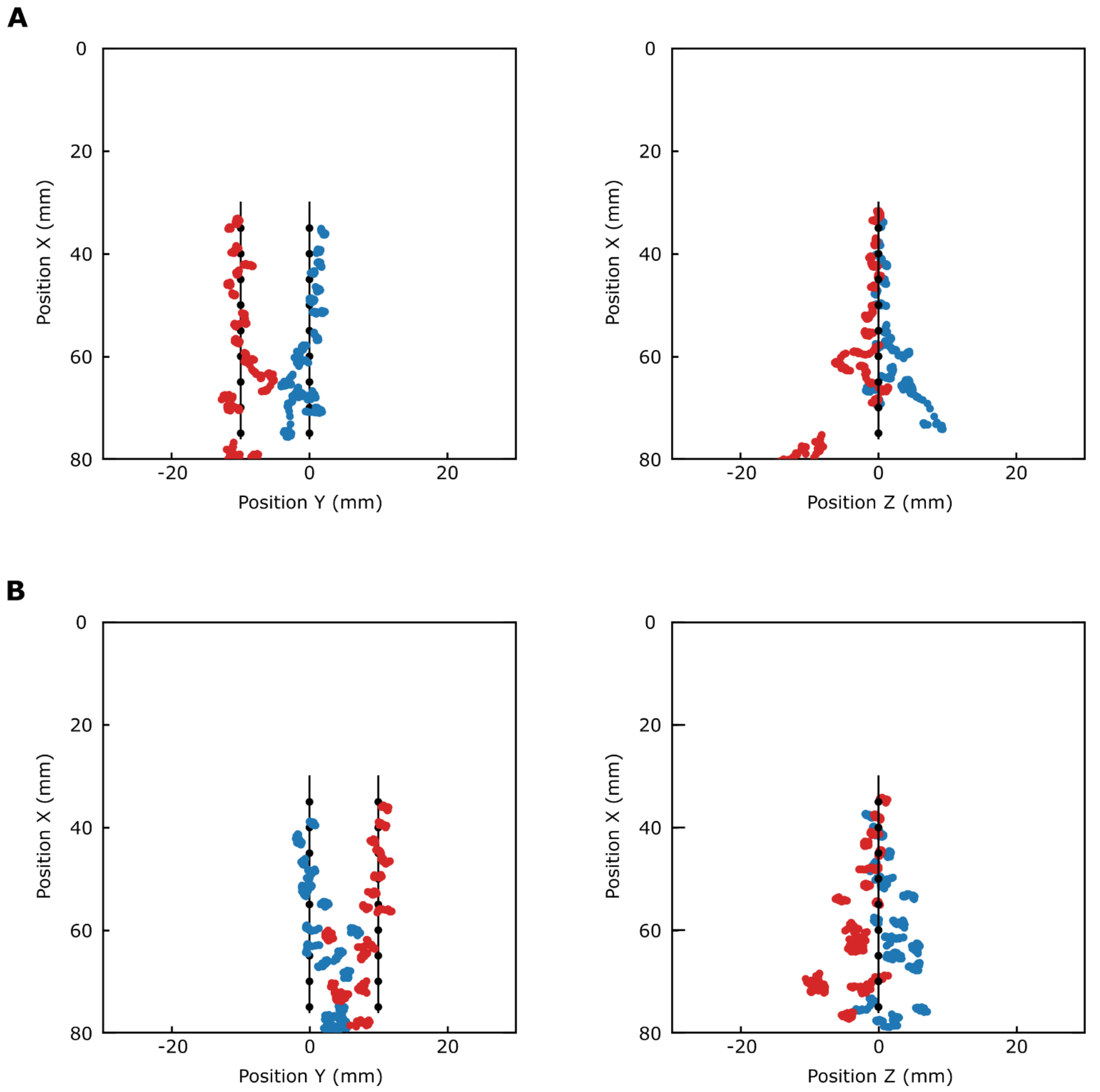

Supplementary Figure 25. Multi-bead experiment with a small distance between trackers. The position estimation accuracy of two trackers at a distance of 1 cm in the left (A) and right (B) planes is investigated.

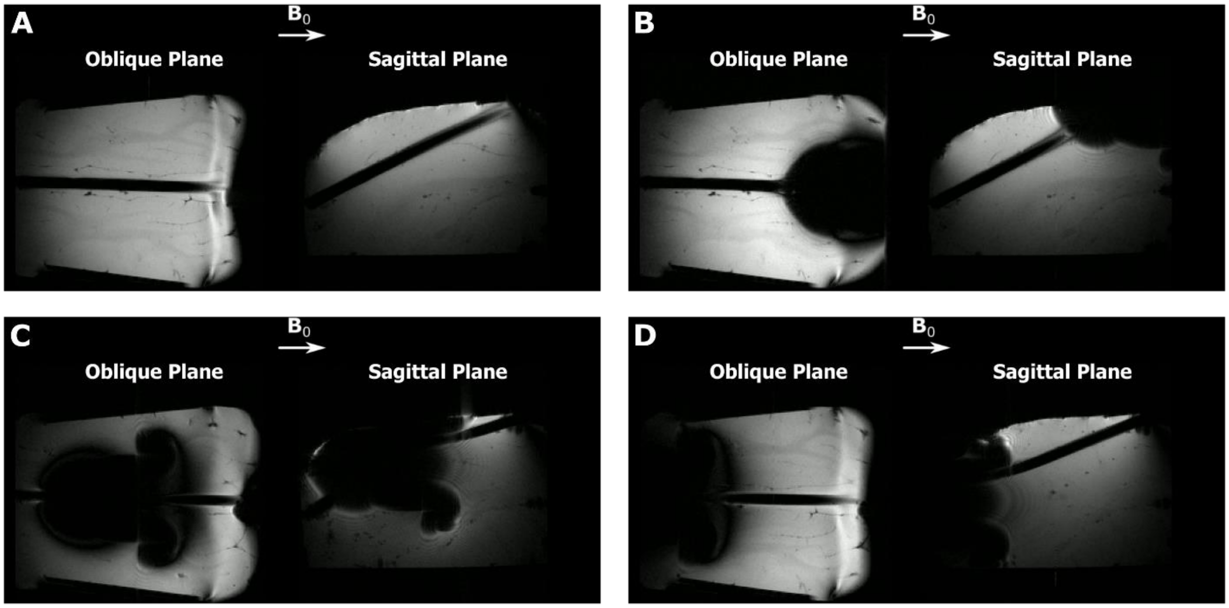

Supplementary Figure 26. Tracker MR images during insertion inside a porcine brain. Tracker artifacts during the insertion inside a porcine brain in oblique and sagittal planes.

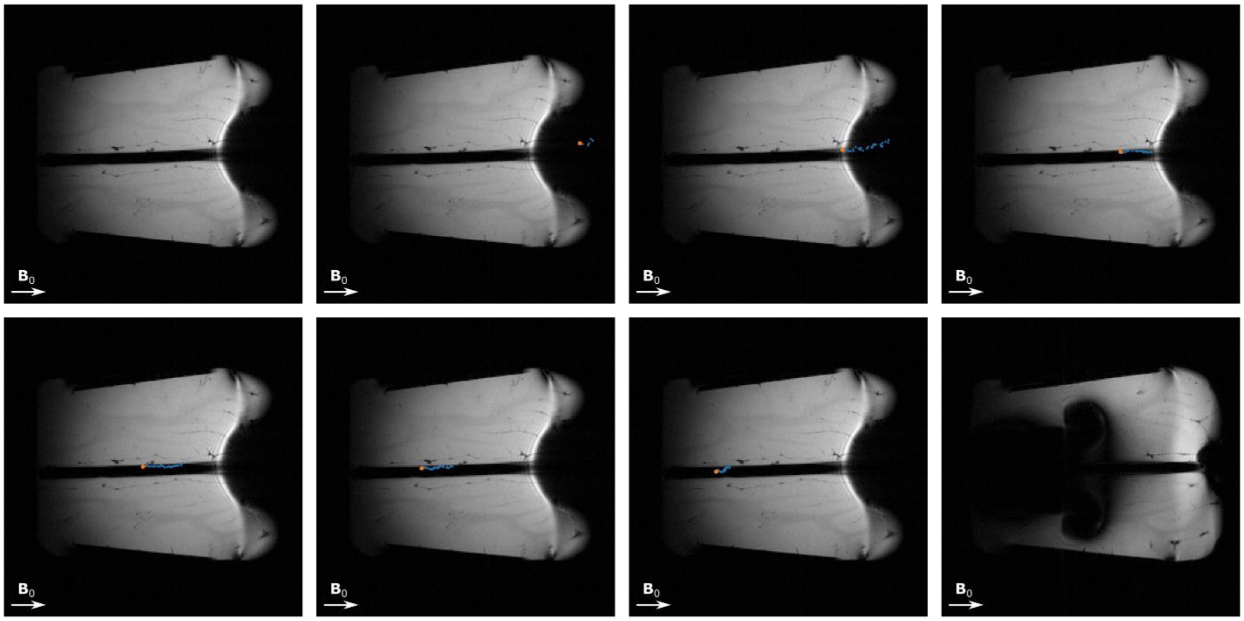

Supplementary Figure 27. Tracker navigation inside a porcine brain with pre- and postoperative images.

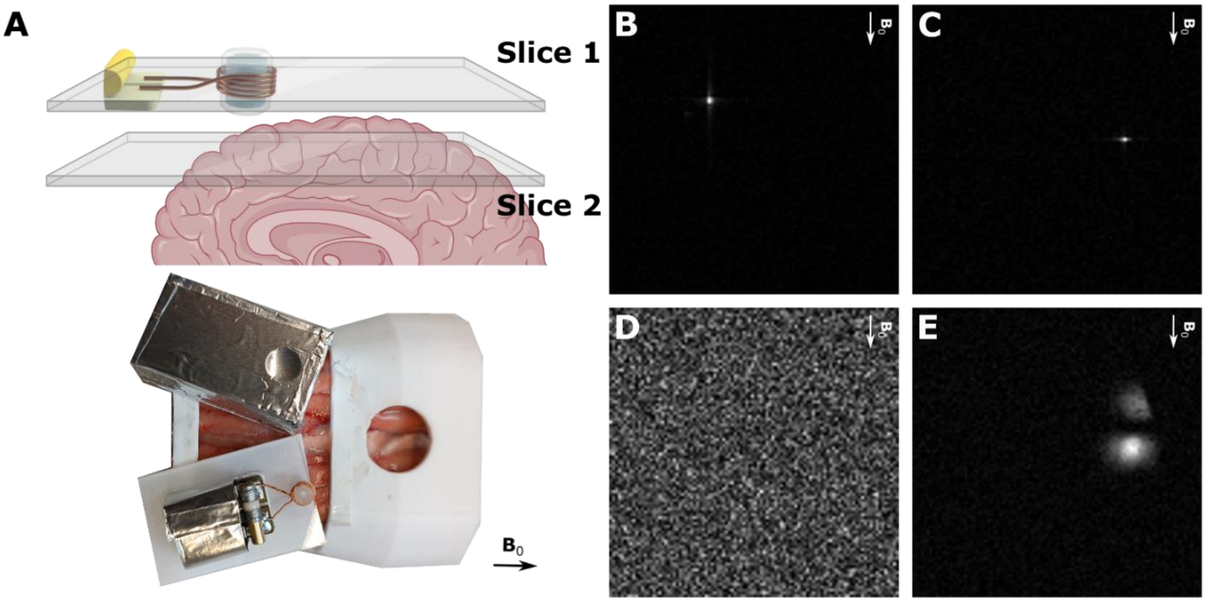

Supplementary Figure 28. **NMR sensor interference with the environment**. A) The setup used to study the extent of the captured NMR signal with and without shielding. B-C) Sensor head image in the upper coronal slice (slice 1) with the shielded sensor (B) and the unshielded sensor. D-E) Image of the lower coronal slice (slice2) with the shielded sensor (D) and unshielded sensor (E).

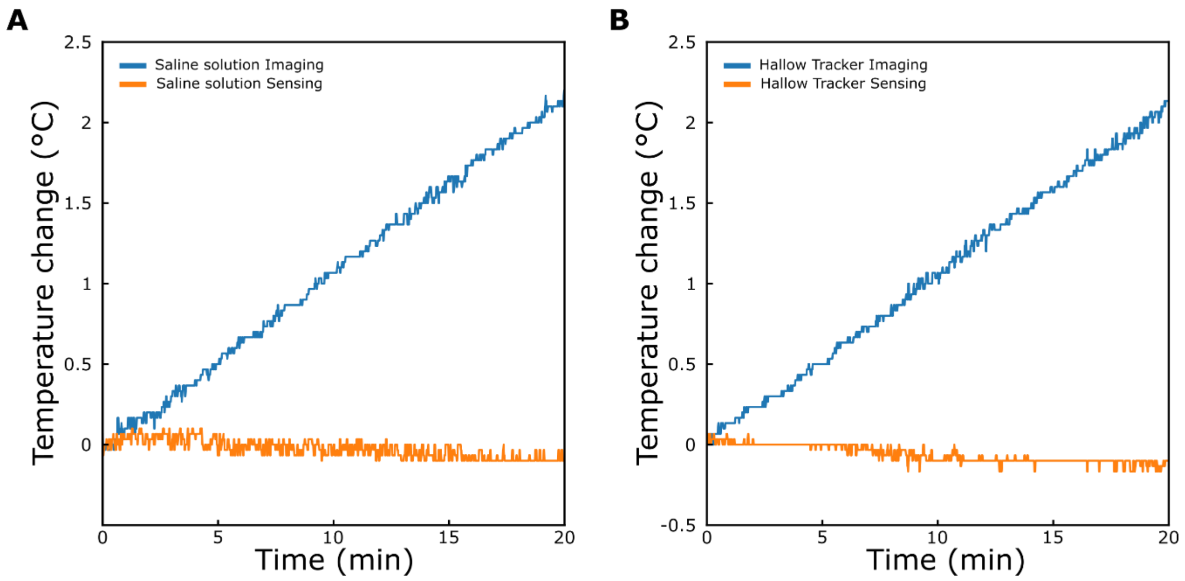

Supplementary Figure 29. **Rf heating comparison in imaging and sensing mode inside MRI**. Temperature measurement of 80 ml of 2.6 M saline solution over 20 minutes (A) and with a hollow tracker inside the solution (B) in imaging and sensing modes.

Supplementary References

1. Bloch, F., Hansen, W. W. & Packard, M. The Nuclear Induction Experiment. Physical Review 70, 474–485 (1946).

2. Tiryaki, M. E., Elmacioğilu, Y. G. & Sitti, M. Magnetic guidewire steering at ultrahigh magnetic fields. Science Advances 9, 1–16 (2023).

3. Beleggia, M., Vokoun, D. & De Graef, M. Demagnetization factors for cylindrical shells and related shapes. J Magn Magn Mater 321, 1306–1315 (2009).

4. Brown, I. J., Bird, J. M., McDougall, I. L. & Black, D. Magnet assembly for use in NMR apparatus. (1986).

5. Son, D., Yim, S. & Sitti, M. A 5-D Localization Method for a Magnetically Manipulated Untethered Robot Using a 2-D Array of Hall-Effect Sensors. IEEE/ASME Transactions on Mechatronics 21, 708–716 (2016).

6. Gleich, B., Schmale, I., Nielsen, T. & Rahmer, J. Miniature magneto-mechanical resonators for wireless tracking and sensing. Science 380, 966–971 (2023).

7. Sharma, S. et al. Location-aware ingestible microdevices for wireless monitoring of gastrointestinal dynamics. Nature Electronics 6, 242–256 (2023).

8. Osaki, Y., Hashi, S., Yabukami, S., Kanetaka, H. & Ishiyama, K. Wireless Magnetic Position-Detection System. IEEE Sensors Journal 17, 4412–4419 (2017).

9. Von Arx, D. et al. Simultaneous Localization and Actuation Using Electromagnetic Navigation Systems. IEEE Transactions on Robotics 40, 1292–1308 (2024).
