# Supplementary material for: Nuclear magnetic resonance for wireless magnetic tracking
Source: Nat Commun. 2025 Dec 2;16:10840. doi: 10.1038/s41467-025-66468-3 (PMC12673108; doi:10.1038/s41467-025-66468-3)
Supplement: Supplementary file 2 — Description of Additional Supplementary Files [file 41467_2025_66468_MOESM2_ESM.pdf]

Description of Additional Supplementary Files

Supplementary Movie 1: Magnetic measurement of an external moving dipole. The magnetic field inside the MRI is recorded while a huge magnet is moved near the entrance of the scanner.

Supplementary Movie 2: 3D visualisation of the precision map in the head, thigh, and abdominal region.

Supplementary Movie 3: Catheter tracking in an ex vivo experiment with a porcine brain. The overlayed tracking estimation with preoperative and postoperative MR images.

Supplementary Movie 4: Ex vivo experiment with a porcine brain. MR images of the catheter insertion process inside a porcine brain are compiled into a video for better visualization.

Supplementary Movie 5: Optic fiber tracking experiment. A hollow magnet is used as the tracker and, combined with the laser as its core, is moved inside a spiral channel.

Supplementary Movie 6: Endoscope camera tracking inside the porcine oesophagus. The live 3D position estimation of the endoscope tip is presented, accompanied by a camera feed.

Supplementary Movie 7: Flexible tracker experiment. The flexible tracker is moved inside complex channels.

Supplementary Movie 8: Balloon catheter inflation-deflation experiment. The balloon catheter is filled with iron oxide particles inside the MRI.

Supplementary Movie 9: Balloon catheter tracking experiment. The inflated balloon catheter is moved inside a channel, and a blue dot shows the estimated position.

Supplementary Movie 10: MRI-based tracking vs NMR tracking sound comparison. The recorded sounds of MRI while functioning in different tracking modes are compared.
